# Familial Frontal Fibrosing Alopecia Occurs Early in Daughters With Affected Mothers: A Case Report and a Review of the Literature

**DOI:** 10.1111/ajd.14463

**Published:** 2025-04-01

**Authors:** Adrienne Oxenham, Annabel Stevenson

**Affiliations:** ^1^ Faculty of Health and Medical Sciences The University of Adelaide Adelaide South Australia Australia; ^2^ Department of Dermatology Royal Adelaide Hospital Adelaide South Australia Australia; ^3^ Department of Dermatology Queen Elizabeth Hospital Adelaide South Australia Australia; ^4^ Department of Dermatology Women's and Children's Hospital Adelaide South Australia Australia

## Abstract

Frontal fibrosing alopecia (FFA) is a form of cicatricial alopecia that is being increasingly diagnosed in recent years. It predominantly affects post‐menopausal women of various ethnic backgrounds, but cases have also been reported in pre‐menopausal women and rarely in men. Eleven familial cases of FFA have been published in the literature, with some authors raising speculation about potential genetic predisposition or shared exposure to environmental factors as triggers for the condition. Despite this, there remains a lack of detailed characterisation of the clinical features specific to familial cases of FFA. In this report, we aim to contribute to the understanding of FFA by presenting the first Australian case of familial FFA involving a mother and her daughter, while also attempting to define the clinical distinguishing features in familial FFA and sporadic FFA.

## 1 Introduction

Frontal fibrosing alopecia (FFA) is a form of progressive scarring alopecia presenting with frontal and temporoparietal recession of the hairline [1]. Initially described by Kossard [2] in 1994 among postmenopausal women, its incidence has risen, affecting not only postmenopausal women but also premenopausal women and, on rare occasions, men. The band‐like frontal hairline recession may extend laterally, moving upwards and behind the ears [2]. Its progression can vary, either occurring gradually or rapidly, or it may remain limited in its extent. Loss of eyebrows (often described as the first sign of disease in most patients) can be either complete or partial [1]. In some cases, FFA can also be linked to the loss of body hair. Although the aetiology of FFA remains unknown, genetic predisposition and shared exposure to environmental factors may contribute to the development of the disease.

The first case of familial FFA was first reported by Roche in 2008, occurring in a sister and brother [3]. After conducting a thorough systematic analysis of the existing scientific literature on this topic, we identified a total of 11 articles describing familial FFA, of which this report is the twelfth. In this review, we report a case of familial FFA involving a mother and daughter. We provide insights into their clinical features, examination findings, and key demographic information. We also attempt to compare our findings from the systematic analysis of reported familial FFA cases to those of the general population with FFA.

## 2 Case Report

A 65‐year‐old postmenopausal woman reported a 16‐year‐long gradual recession of her frontotemporal hairline and loss of eyebrows. Following examination, she was diagnosed with Frontal Fibrosing Alopecia (FFA). Interestingly, she noted similar symptoms in her mother. Three months later, the 65‐year‐old woman returned for a follow‐up with her 88‐year‐old mother. Furthermore, the woman's two daughters, aged 31 and 34, were also present and examined but did not display any signs of FFA at that time. We summarise the clinical history of each patient below.

### 2.1 Patient 1

An 88‐year‐old woman of Italian ethnicity sought an initial consultation after her daughter was diagnosed with Frontal Fibrosing Alopecia (FFA) a few months earlier. Symptoms arose at the age of 60 when her hairline started receding. Gradually, loss of her hairline, eyebrows, and body hair on the arms and legs was noted, accompanied by pruritus. Past medical history included a hysterectomy at the age of 40 for menorrhagia, rheumatoid arthritis, and Autoimmune Thyroid Disease. She took iron supplements and had previous exposure to oral contraceptive pills for a few years. Significant emotional stress provoked by the loss of a spouse and a stroke predated the onset. Family history included similar symptoms in her sister (who was not present for examination) and daughter (patient 2).

**FIGURE 1:**
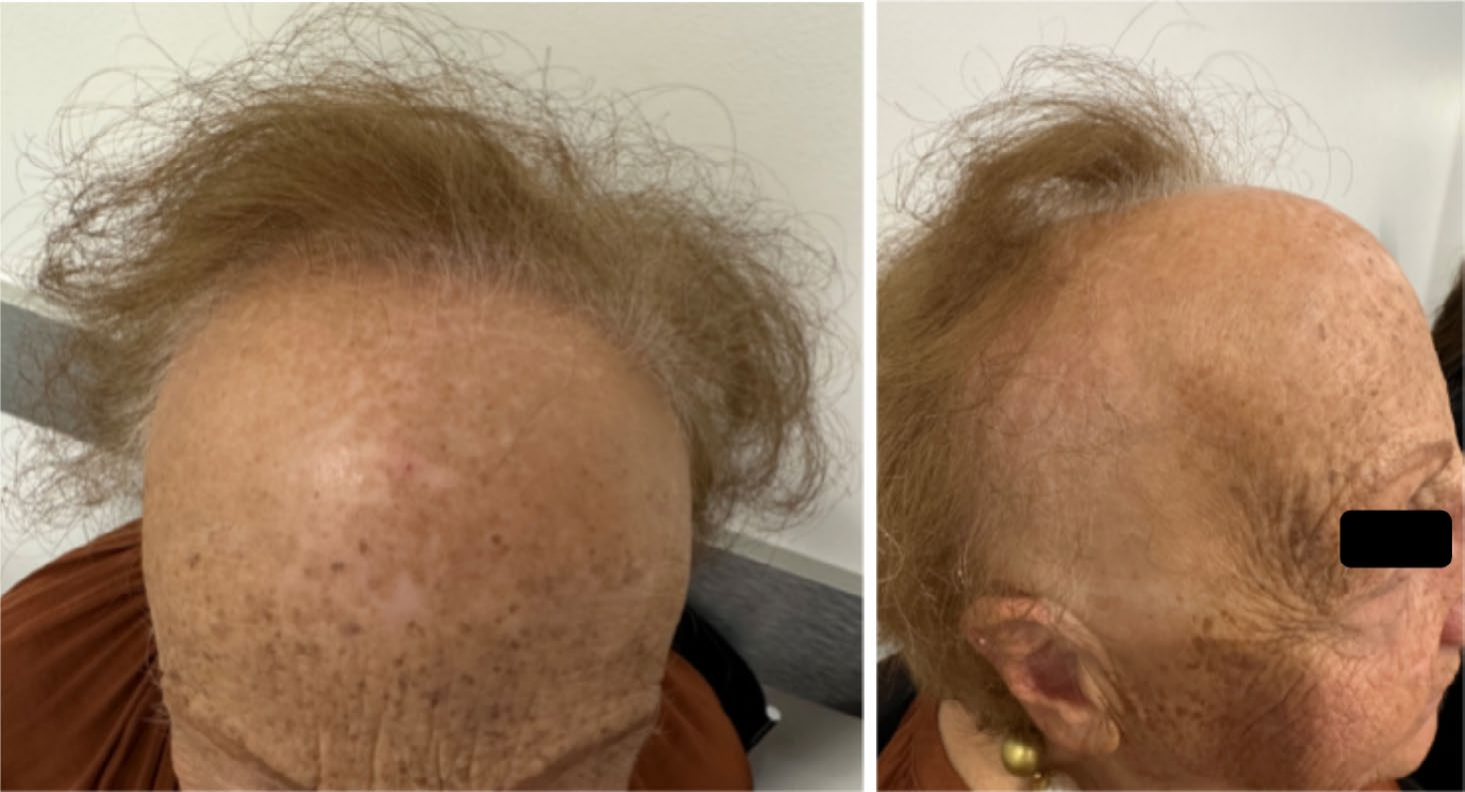
Severe frontal recession and eyebrow loss affecting patient 1 (mother).

**FIGURE 2:**
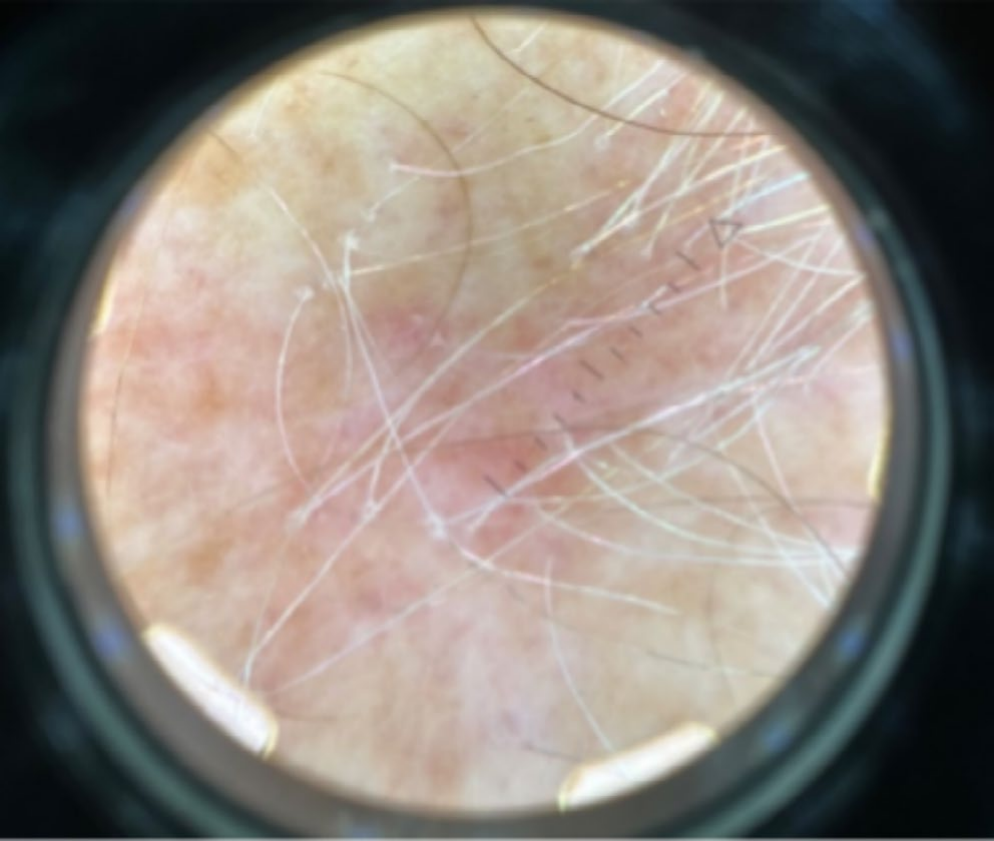
Trichoscopic examination showing perifollicular scale.

Physical examination exhibited a glabella to hairline measurement of 10.2 cm, loss of eyebrows (Figure 1), and body hair on her arms and legs. No facial papules were observed, but trichoscopic examination revealed perifollicular scale (Figure 2).

### 2.2 Patient 2

The 65‐year‐old daughter of patient 1, also of Italian ethnicity, received a clinical diagnosis of FFA after presenting with the loss of eyebrows, then frontotemporal hair loss, and body hair loss on her axilla, arms, and legs. Facial papules were observed. She had a history of exposure to the oral contraceptive pill (OCP) for 12 months and hormone replacement therapy (HRT) for 12 months, discontinued due to migraines. Her medical history included six recurrent miscarriages (no cause identified), multiple transient ischaemic attacks (TIAs), hysterectomy for adenomyosis, and hypertension. Regular medications included irbesartan and pantoprazole. Family history included her mother (patient 1) and maternal aunt suffering from a similar presentation.

**FIGURE 3:**
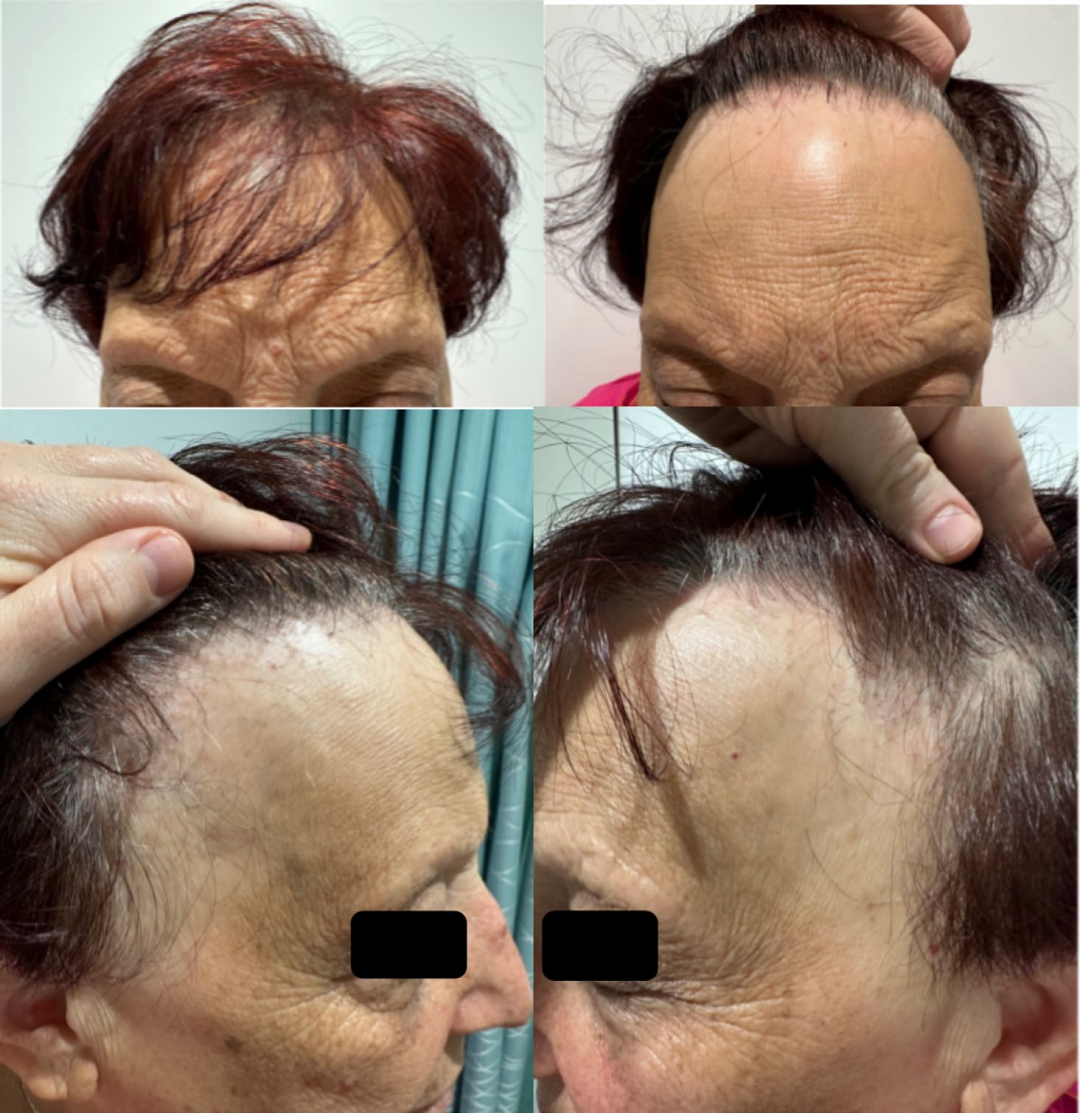
Frontal recession and eyebrow loss affecting patient 2 (daughter).

**FIGURE 4:**
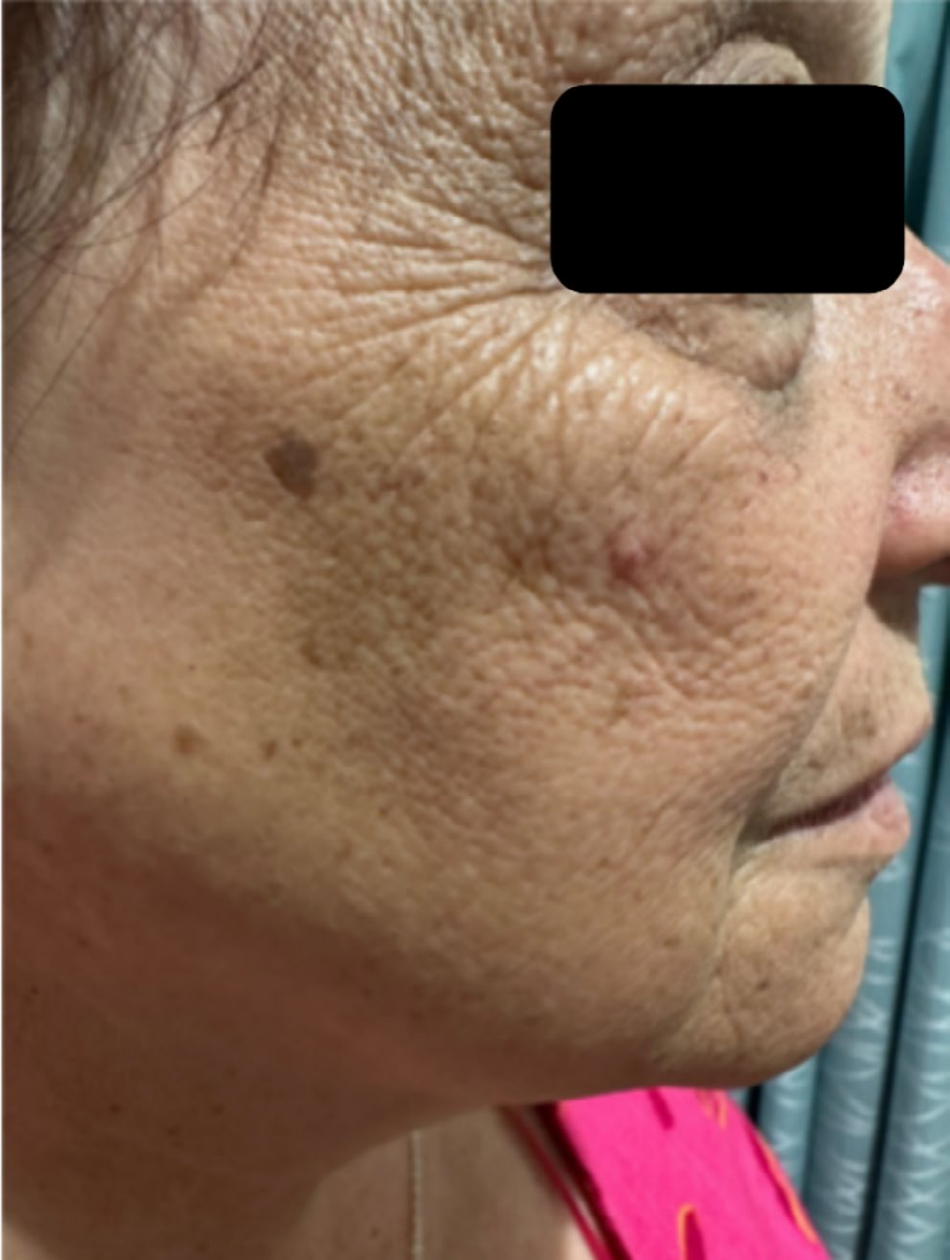
Facial papules (patient 2).

**FIGURE 5:**
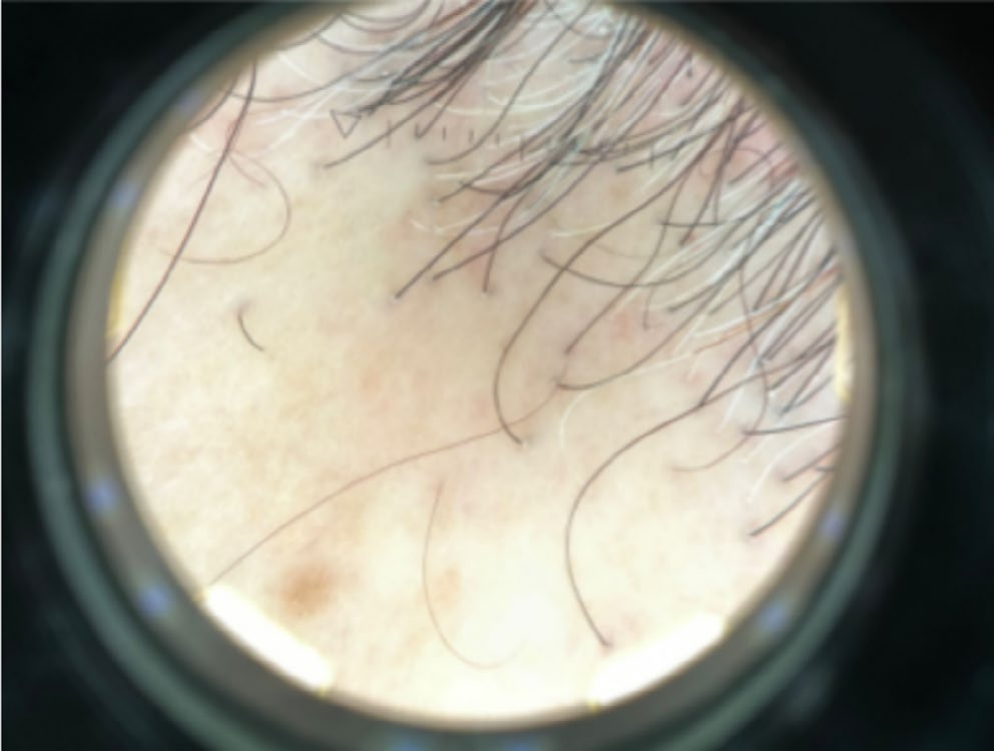
Trichoscopic examination showing perifollicular scaling, reduced hair density, and the lonely hair sign.

Examination demonstrated eyebrow loss, frontotemporal hairline recession with a glabella to hairline measurement of 7.2 cm (Figure 3), body hair loss in the axilla, arms, and legs, as well as facial papules (Figure 4). Trichoscopy revealed perifollicular scaling, decreased hair density, the lonely hair sign, and areas of scarring alopecia at the vertex clinically consistent with Lichen Planopilaris (Figure 5).

## 3 Methods

A PubMed literature search was conducted using the terms “Familial Frontal Fibrosing Alopecia” and “Familial FFA Case Report”. We included a total of 12 reported articles in our analysis, including this one. A total of 36 cases of familial FFA were counted within the 12 articles. After a systematic analysis, clinical and demographic data have been summarised from these patients, as well as our new case, outlined in Tables 1 and 2. Additionally, differences were compared between FFA within the general population and familial FFA.

**TABLE 1:** Summary of clinical cases of FFA in the literature.

Reference		Ethnicity	Relation	Age	Pre/post‐menopausal	Age at menopause	Duration (months)	Eyebrow	Frontotemporal hair loss	Body hair loss	Associated disease
Cuenca‐Barrales et al., 2021 [4]	3F	Spanish	Sisters	62	Post‐menopausal	52	12	Yes	Yes	No	Cutaneous Lupus, Breast cancer
				67	Post‐menopausal	53	120	Yes	Yes	No	
				59	Post‐menopausal	50	36	No	Yes	No	
Gavazzoni Dias et al., 2023 [5]	2F	Black	Mother	76	Post‐menopausal	Unknown	120	Yes	Yes	No	
			Daughter	45			12	Yes	Yes	No	
	2F	Caucasian	Mother	74	Post‐menopausal	Unknown	Unknown	Yes	Yes	No	
			Daughter	49	Pre‐menopausal		24	Yes	Yes	No	
	2F	Caucasian	Mother	84	Post‐menopausal	Unknown	144	Yes	Yes	No	
			Daughter	61	Post‐menopausal	Unknown	72	Yes	Yes	Yes	
	2F	Unknown	Mother	70	Post‐menopausal	Unknown	60	Yes	Yes	No	
			Daughter	42	Pre‐menopausal		24	Yes	Yes	No	
	2F	Caucasian	Mother	72	Post‐menopausal	Unknown	Unknown	Yes	Yes	No	
			Daughter	37	Pre‐menopausal		Unknown	Yes	Yes	No	
Dlova et al., 2012 [6]	3F	Italian/Caucasian	Sisters	55	Post‐menopausal	Unknown	12	Yes	Yes	No	
				61	Post‐menopausal	Unknown	24	Yes	Yes	No	
				73	Post‐menopausal	Unknown	Unknown	Yes	Yes	No	
	1F	Italian/Caucasian	Sister	59	Post‐menopausal	Unknown	48	Yes	No	Yes	Lichen Planopilaris
	1M		Brother	62			12	Yes	No	Yes	
	2F	Spanish	Sisters	25	Pre‐menopausal		12	Yes	No	Yes	
		Spanish		21	Pre‐menopausal		3	Yes	No	Yes	
	3F	Black	Mother	74	Post‐menopausal	Unknown	24	No	Yes	No	
			Daughter	50	Pre‐menopausal		12	No	Yes	No	
			Cousin	44	Pre‐menopausal		12	Yes	No	No	
	1F	Unknown	Sister	75	Post‐menopausal	Unknown	12	Yes	No	No	

	1M	Unknown	Brother	71			8	Yes	No	No	
	2F	Italian/Caucasian	Sisters	57	Post‐menopausal	Unknown	12	Yes	No	No	
				59	Post‐menopausal	Unknown	12	Yes	No	No	
	2F	Unknown	Twin Sisters	67	Post‐menopausal	Unknown	12	Yes	No	Yes	Vitiligo
			67	Post‐menopausal	Unknown	6	Yes	No	Yes	Vitiligo
Rocha et al., 2020 [7]	6F	Unknown	Sisters	52	Post‐menopausal	Unknown	Unknown	Yes	Yes	Yes	Hypertension, Diabetes Mellitus
				62	Post‐menopausal	48	Unknown	Yes	Yes	No	Hysterectomy
				60	Post‐menopausal	55	Unknown	No	No	Yes	
				31	Pre‐menopausal		Unknown	Yes	No	Yes	Allergic Rhinitis
				50	Post‐menopausal	49	Unknown	Yes	No	Yes	Deep Vein Thrombosis
				49	Post‐menopausal	Unknown	Unknown	Yes	No	No	
Navarro‐Belmonte et al., 2015 [1]	2F	Unknown	Mother	75	Post‐menopausal	45	Unknown	Yes	Yes	No	Hypothyroidism
		Unknown	Daughter	47	Pre‐menopausal		Unknown	Yes	Yes	No	Psoriasis, Hypertension, Dyslipidaemia Type 2 Diabetes Mellitus, Hypertension
	2F	Unknown	Mother	60	Post‐menopausal	53	Unknown	Yes	Yes	No	
		Unknown	Daughter	41	Pre‐menopausal		Unknown	Yes	Yes	No	Primary Biliary Cirrhosis
	2F	Unknown	Mother	63	Post‐menopausal	48	Unknown	Yes	Yes	No	
		Unknown	Daughter	37	Pre‐menopausal		Unknown	Yes	Yes	No	Seborrheic Dermatitis
	2F	Unknown	Mother	62	Post‐menopausal	55	Unknown	Yes	Yes	No	
		Unknown	Daughter	33	Pre‐menopausal		Unknown	Yes	No	No	
Porriño‐Bustamante et al., 2019 [1]	2M	Unknown	Brothers	44			48	Yes	Yes	Yes	Excision of Pituitary macroadenoma leading to panhypopituitarism
		Unknown		46			12	Yes	Yes	Yes	
	2M	Unknown	Son	27			42	Yes	No	No	
		Unknown	Father	60			Unknown	Yes	Yes	No	
Ocampo‐Garza et al., 2021 [8]	2F	Unknown	Mother	72	Post‐menopausal	Unknown	Unknown	Yes	Yes	Yes	Hysterectomy, Rheumatoid Arthritis
		Unknown	Daughter	51	Pre‐menopausal	Unknown	Unknown	Yes	Yes	Yes	Lichen planus pigmentosus
	2F	Unknown	Mother	58	Post‐menopausal	Unknown	Unknown	Yes	Yes	Yes	Lichen planus pigmentosus, Diabetes Mellitus, Hypertension
		Unknown	Daughter	39		Unknown	Unknown	No	Yes	No	Hysterectomy, Hypothyroidism
	2F	Unknown	Sister	69	Post‐menopausal	Unknown	Unknown	Yes	Yes	Yes	
		Unknown	Sister	75	Post‐menopausal	Unknown	Unknown	Yes	Yes	Yes	
Junqueira Ribeiro Pereira et al., 2010 [9]	2F	Unknown	Sister	65	Post‐menopausal	Unknown	Unknown	No	Yes	No	
		Unknown	Sister	59	Post‐menopausal	Unknown	12	No	Yes	No	Oophorectomy, colorectal cancer, chemotherapy, Hepatitis C
Cranwell and Sinclair, 2017 [10]	2F	Caucasian	Daughter	46	Pre‐menopausal		10	No	Yes	No	Relapsing and remitting alopecia arerata, Rheumatoid Arthritis
		Caucasian	Mother	Unknown	Post‐menopausal	Unknown	Unknown	Yes	Yes	No	Scleroderma
Roche, 2008 [3]	1F	Unknown	Sister	75	Post‐menopausal	Unknown	12	Yes	Yes	No	
	1M	Unknown	Brother	71			9	Yes	Yes	No	
Porriño‐Bustamante et al., 2018 [11]	2F	Caucasian	Sisters	62	Post‐menopausal	50	36	Yes	Yes	Yes	
		Caucasian		66	Post‐menopausal	54	36	Yes	Yes	Yes	Androgenic Alopecia
	3F	Caucasian	Mother	78	Post‐menopausal	55	48	Yes	Yes	Yes	
		Caucasian	Cousin of mother	68	Post‐menopausal	52	48	Yes	Yes	Yes	Androgenic Alopecia
		Caucasian	Daughter	50			36	Yes	Yes	Yes	
	2M	Caucasian	Brothers	44			48	Yes	Yes	Yes	
		Caucasian		46	Pre‐menopausal		12	Yes	Yes	Yes	Lichen planopilaris with scarring alopecia
	2F	Caucasian	Sisters	39	Pre‐menopausal		72	Yes	Yes	Yes	
		Caucasian		42	Pre‐menopausal		24	Yes	No	No	
	3F	Caucasian	Sister	88	Post‐menopausal	50	24	Yes	Yes	Yes	
		Caucasian	Sister	72	Post‐menopausal	50	12	Yes	Yes	No	
		Caucasian	Niece	64	Post‐menopausal	50	48	Yes	Yes	Yes	
	2F	Caucasian	Sisters	53	Post‐menopausal	49	7	Yes	Yes	No	
		Caucasian		46	Pre‐menopausal		12	Yes	Yes	No	
	2F	Caucasian	Sisters	74	Post‐menopausal	39	288	Yes	Yes	Yes	
		Caucasian		72	Post‐menopausal	51	240	Yes	Yes	Yes	
	2F	Caucasian	Sisters	77	Post‐menopausal	52	204	Yes	Yes	Yes	
		Caucasian		74	Post‐menopausal	50	48	Yes	Yes	Yes	
	2F	Caucasian	Mother	70	Post‐menopausal	51	96	Yes	Yes	No	
		Caucasian	Daughter	44	Pre‐menopausal		24	Yes	Yes	No	
Current Case	2F	Italian/Caucasian	Mother	88	Post‐menopausal	40	336	Yes	Yes	Yes	Rheumatoid Arthritis, Autoimmune thyroid disease, Stroke
		Italian/Caucasian	Daughter	65	Post‐menopausal	Unknown	192	Yes	Yes	Yes	Recurrent Miscarriages, Multiple TIAs

**TABLE 2:** Grouping of the most common demographic and clinical details of familial FFA.

Demographics and clinical characteristics in reported familial FFA cases in literature	
Demographics	Results
Age at diagnosis (mean)	58.3
Gender	72 Female (88.9%) 9 Male (11.1%)
Post‐menopausal	52 (64.2%)
Pre‐menopausal	20 (24.7%)
Age at menopause (mean)	50.0
Ethnicity	28 Caucasian (34.5%) 5 Spanish (6.2%) 9 Italian/Caucasian (11.1%) 5 African Descent (6.2%) 34 Unknown (42.0%)
Relationships	14 Sister and sister (38.8%) 16 Mother and daughter (44.4%) 3 Brother and sister (8.3%) 2 Brother and brother (5.5%) 1 Father and son (2.7%)
Eyebrow loss	73 (90.1%)
Frontotemporal hair loss	63 (77.7%)
Body hair loss	34 (42.0%)
Other sites of alopecia	Axillae Beard Limbs Eyelashes Pubic Hair
Dermoscopy findings	40 Perifollicular erythema/scale (49.4%) 17 Lonely hair sign (21%) 6 Lichen planus pigmentosus (7.4%) 24 Absence of vellus hair (29.6%)
Other associated symptoms	18 Facial papules (22.2%)
Associated diseases	5 Hypertension 3 Diabetes mellitus 1 Syslipidaemia 3 Hysterectomy 1 Lupus 2 Vitiligo 1 Deep vein thrombosis 1 Allergic rhinitis 3 Thyroid disease 1 Psoriasis 1 Seborrheic dermatitis 2 Rheumatoid arthritis 1 Oophorectomy 1 Breast cancer 1 Colorectal cancer 1 Pituitary macroadenoma 1 Primary biliary cirrhosis 1 Hepatitis C 1 Recurrent miscarriage

## 4 Discussion

Our analysis revealed that while familial FFA predominantly impacts postmenopausal women (64.2%), it also occurred in premenopausal women (24.7%) and less frequently in men (9%). Within the affected families, the mean age at menopause was 50 years (range 39–55). A review estimated the mean age at diagnosis for FFA to be between 56 and 63 years old in the general population [13]. However, in our review of familial FFA cases, the age at diagnosis ranged from 21 to 88 years, with the younger affected patients primarily being daughters. This suggests that daughters of affected mothers may be more likely to develop the condition at an earlier age compared to women in the general population. Further research is required to confirm whether this trend is driven by genetic predisposition, environmental exposure, or lead‐time bias (where daughters of affected mothers may recognise early symptoms sooner than others).

An analysis of familial connections revealed that the most prevalent relationship was between mother and daughter (44.4%), followed by sister and sister (38.8%) and brother and sister (8.3%). Among the 16 mother–daughter pairs, it was observed that all mothers were postmenopausal at the time of diagnosis. In contrast, the majority of daughters were premenopausal, with only three having undergone surgical menopause. In terms of ethnicity, the majority of families with a specified background were Caucasian (51.9%) with the largest single ethnic group being Italian (11.1%) followed by Spanish descent at 6.2%. Additionally, 6.2% of patients were Black. While this distribution may reflect publication bias, it remains a noteworthy finding that warrants further investigation. These findings correlate with trends observed in FFA cases in the general population, where the condition is predominantly reported among Caucasians [13].

In our review of familial FFA, 77.7% were affected by frontotemporal hair loss. Eyebrow loss was a feature in 90.1% of affected families, compared to 63%–83% [13] of unrelated FFA cases in previous studies [13]. Body hair loss, including axillae, beard, limbs, eyelashes, and pubic hair, was found in 42% of familial FFA cases, whereas reported rates in general FFA cases range from 22%–77% [13]. Findings of facial papules due to vellus hair involvement were similar in both subtypes of FFA, with a higher prevalence in premenopausal patients. Other symptoms and clinical findings commonly described in general FFA but rarely documented in familial cases include pruritus, increased preauricular lines, excessive scalp sweating, and follicular re‐pigmentation of the white/grey hair along the frontal, temporal, and occipital hairline [13]. While these findings are noteworthy, it is important to consider the potential underreporting of symptoms such as eyebrow and body hair loss in both patient groups. More comprehensive documentation of both positive and negative symptoms, along with statistical validation, would help refine our understanding of the clinical distinctions between these groups.

Common comorbidities in both familial and non‐familial FFA included hypertension, diabetes mellitus, thyroid disease, and autoimmune disorders such as rheumatoid arthritis, lupus, and vitiligo. Hysterectomy seems to be a common trigger in both sporadic and familial FFA, especially when women had to go through an early surgical menopause. There was a high prevalence of rosacea in severe cases of sporadic FFA (15%–61%) [13]; however, none were recorded in our analysis of familial FFA cases. The absence of rosacea in familial cases may be due to underreporting rather than a true difference. Likewise, atopy was observed in 43.9% of non‐familial FFA cases but was rarely reported in familial FFA. Further studies comparing these factors are necessary before drawing definitive conclusions.

While the demographic profiles of FFA and familial FFA do not exhibit significant disparities, our study revealed a significant trend among mother–daughter pairs: all mothers were postmenopausal at the time of diagnosis, while every daughter except for three who had undergone a hysterectomy was premenopausal (Table 3).

**TABLE 3:** Proposed clinical distinguishing features in familial FFA.

Feature	Familial FFA	Sporadic FFA	Comments
Age of onset	Mostly in postmenopausal women (64.2%). However, increased likelihood of premenopausal diagnosis (24.7%), especially in daughters of affected mothers. (Table 2)	Typically, postmenopausal onset occurs in 83%–95% of cases [13]	Requires further study to confirm genetic/environmental factors
Eyebrow loss	Reported in 90.1% of cases. (Table 2)	Reported in 63%–83% of cases [13]	Needs statistical validation; possible underreporting in non‐familial cases
Body Hair Loss	Reported in 42% of cases. (Table 2)	Reported in 22%–77% of cases [13]	Variability suggests potential underreporting
Rosacea and atopy	No reported cases of rosacea. 1 reported case of allergic rhinitis. (Table 1)	Reported in up to 15%–61% and 43.9% of cases, respectively [13]	Likely due to underreporting rather than a true difference
Ethnicity	Largest single ethnic group reported—Italian at 11.1% (Table 2)	More diverse ethnic distribution [13]	Likely influenced by publication bias.

The presence of FFA within families suggests a combination of genetic predisposition and shared exposure to common environmental triggers. Tziotzioz et al. observed a genome‐wide significant association of FFA in four genomic loci. These provided insight into the pathogenesis, highlighting FFA as a genetically predisposed immune‐inflammatory disease driven by HLA‐B*07:02 [14]. In fact, a number of studies found that most of the patients in that cohort shared a specific haplotype, which may predispose them to familial FFA [13].

Dlova and Tosti explored the correlation between environmental factors and the rising prevalence of FFA in their case series on familial FFA. They proposed that the manifestation of the condition among family members could suggest shared exposure to common environmental triggers [6] such as the use of the same topical agents among family members. This proposition gains support from Trüeb and da Silva Libório's documentation of the initial case of connubial FFA in a genetically unrelated couple [15]. Consequently, evaluating the hair condition and grooming practices of marital partners becomes crucial in identifying potential environmental triggers.

With regards to the data summarised in Table 3, further research is required to delineate whether the wider age range at diagnosis—particularly the younger age of onset in daughters—is due to some genetic predisposition, environmental exposure, or lead time bias (as daughters who are aware of their mothers being afflicted are more likely to recognise subtle early clinical signs that may otherwise be overlooked). Additionally, publication bias may lead to increased recognition of this condition among those of Italian background. Similarly, the lack of reported cases of rosacea and atopy in familial FFA may be due to underreporting rather than a true difference.

## 5 Conclusion

In conclusion, our summary entails a case report detailing familial FFA in a mother‐daughter pair, complemented by a comprehensive review of existing literature on familial FFA. While some distinguishing features between familial and non‐familial FFA have been identified (Table 3), further research with a larger cohort is essential to clarify genetic contributions and external environmental triggers. Ongoing research will enhance our understanding of FFA, leading to improved early identification and prognosis. Our findings suggest that mothers diagnosed with FFA should inform their daughters about early signs of the disease to facilitate prompt diagnosis and prevent progression of scarring alopecia.

## Conflicts of Interest

The authors declare no conflicts of interest.

## Data Availability

The data that support the findings of this study are openly available in PubMEd at https://pubmed.ncbi.nlm.nih.gov.
